# Knowledge, attitudes, and practices of veterinary professionals towards ticks and tick-borne diseases in Illinois

**DOI:** 10.1016/j.onehlt.2022.100391

**Published:** 2022-04-24

**Authors:** Samantha D. Crist, Heather Kopsco, Alexandria Miller, Peg Gronemeyer, Nohra Mateus-Pinilla, Rebecca L. Smith

**Affiliations:** a Department of Pathobiology, College of Veterinary Medicine, University of Illinois Urbana-Champaign, USA; b College of Veterinary Medicine, University of Missouri, USA; c Illinois Natural History Survey, Prairie Research Institute, University of Illinois Urbana-Champaign, USA; d Carl R. Woese Institute for Genomic Biology, University of Illinois Urbana-Champaign, USA; e Department of Biomedical and Translational Sciences, Carle Illinois College of Medicine, University of Illinois Urbana-Champaign, USA

**Keywords:** Ticks, Tick-borne diseases, Veterinary, TBD, Tick-borne disease, KAP, Knowledge, attitudes, and practices

## Abstract

**Objective:**

A lack of standardized surveillance or reporting of tick-borne diseases (TBDs) in Illinois creates uncertainty for veterinarians regarding TBDs occurring within their practice geography or which TBDs may be encroaching on their area from neighboring territories. Therefore, the objective of this study was to gauge the knowledge, attitudes, and practices of veterinary professionals in Southern and Central Illinois to establish a foundation for targeting educational and outreach programs that address knowledge gaps.

**Sample:**

72 veterinary professionals in Central and Southern Illinois.

**Procedures:**

An online knowledge, attitudes, and practices survey was distributed to veterinary professionals in Southern and Central Illinois. Poisson regression analyses were conducted to determine factors associated with knowledge scores and the estimated number of TBD cases diagnosed.

**Results:**

Knowledge scores were significantly higher among veterinary practitioners with recent (within the last 5 years) training on TBD. The number of cases of TBD diagnosed was higher among those reporting concern about TBD, and among those who routinely test for TBDs. The types of diseases diagnosed were heavily influenced by the diagnostic method used.

**Clinical relevance:**

This study paints a cohesive picture of human factors associated with diagnosing veterinary diseases and TBD prevalence in Southern and Central Illinois. Our results highlight the importance and practical value of veterinary continuing education on ticks and TBDs for both companion animals and public health. Building capacity for training veterinarians in parasitology using partnerships between academia and industry may strengthen the knowledge and understanding of ticks and tick-borne pathogens in the veterinary community.

## 1 Introduction

Encounters with infected ticks are increasingly prevalent across the United States for both humans and companion animals [[1], [2], [3], [4], [5], [6]]. Within the Midwestern United States (USA), Illinois, and particularly the Southern and Central regions, is experiencing encroachment of several tick species [[7], [8], [9]] from the north, south, east, and west of the state.

Multiple studies, including the Illinois Tick Inventory Collaboration Network (I-TICK) program [10], have categorized and tracked tick species throughout Illinois [[11], [12], [13], [14], [15], [16], [17], [18], [19]]. Among these are *Dermacentor variabilis, Ixodes scapularis, Amblyomma maculatum,* and *Amblyomma americanum* [10]. These four tick species commonly parasitize domestic mammals, including dogs and cats. Furthermore, they can transmit tick-borne diseases (TBDs) such as Lyme disease, anaplasmosis, ehrlichiosis, and rickettsiosis [[2], [3], [4], [5]].

Pet ownership is associated with an increased tick encounter risk to humans [6,20]; furthermore, companion animals and humans can be infected by many of the same vector-borne diseases [21,22]. Therefore, in addition to their role in prevention, diagnosis, and treatment of TBDs on companion animals, veterinarians have the unique opportunity to inform pet owners about public health risks associated with ticks and TBDs [23].

Tick-borne diseases of companion and domestic animals currently are not nationally notifiable, and systematic surveillance of these illnesses is lacking [24]. Given this information gap, veterinary practitioners are currently using and applying TBD surveillance data from their human medicine counterparts to their veterinary patients [25]. In 2011, the Companion Animal Parasite Council (CAPC) partnered with IDEXX laboratories to use TBD testing data to produce nationwide prevalence maps that forecast tick-borne pathogen trends, taking into consideration climatological and ecological drivers [24]. While these maps may provide an approximation of potential TBD activity in an area, the data are not always reliable due to a lack of uncertainty measurement for each test, no information on travel or disease history, and highly disproportionate reporting rates [24].

Therefore, while analysis and reporting of human and veterinary tick-borne diseases are mutually beneficial in terms of animal and public health, applying human epidemiological data to veterinary species is an imperfect model [25]. Given these limitations, our understanding of which TBDs are affecting domestic animals in Southern and Central Illinois could be biased since TBDs may present differently and with a varying frequency between species of veterinary interest and humans [25].

Nonetheless, veterinarians play an important role in providing information and promoting awareness about ticks and TBDs of veterinary and medical importance. It is therefore essential to understand the veterinarians' experience with ticks and TBDs to determine how the surveillance for these diseases in animals may be improved, and to identify the efforts that would bolster prevention of TBDs and tick encounters in both humans and their pets. We hypothesized that veterinarians could benefit by gaining knowledge of what TBDs are present in their practice area and which diseases to expect, given a travel history to an area of tick-borne disease status previously unknown to the veterinarian.

The purpose of this study was to examine the knowledge, attitudes, and practices of veterinary professionals in Southern and Central Illinois with regards to ticks and TBDs. This paper seeks to identify factors associated with both knowledge and diagnosis of tick-borne diseases, with the aim of improving veterinary education and continuing education programs on these topics. A secondary aim is to identify how the existing passive surveillance of ticks and TBDs in veterinary practice could be strengthened through these improved education programs.

## 2 Methods

### 2.1 Questionnaire development

An electronic Knowledge, Attitudes, and Practices (KAP) survey [26] was created in REDCap [27] electronic data capture tools hosted at the University of Illinois, Urbana-Champaign, with the support of the Interdisciplinary Health Sciences Institute and Research IT – Technology Services. REDCap (Research Electronic Data Capture) is a secure, web-based application designed to support data capture for research studies, providing: 1) an intuitive interface for validated data entry; 2) audit trails for tracking data manipulation and export procedures; 3) automated export procedures for seamless data downloads to common statistical packages; and 4) procedures for importing data from external sources. The survey was formatted to be mobile-friendly and sent to participants via email. The inclusion criteria were veterinary professionals (including veterinarians, licensed veterinary technicians, and veterinary staff) currently practicing in Central and Southern Illinois. The survey consisted of questions in four domains: demographics, knowledge, attitudes, and practices. Questions based on these concepts were presented in this order and grouped by domain. The demographics section consisted of nine questions, the knowledge section of eighteen questions, the attitude section of nine questions, and the practice section of two questions. Two final questions were included at the conclusion of the survey for a total of 54 questions. Of these 54 questions, 29 were multiple choice, 15.5 were short answer, and 9.5 were select all that apply questions. The survey content was created and question formats were determined by the research team and transferred into REDCap by a single researcher (SC). Beta testing to troubleshoot technical issues was performed by a member of the research team that did not directly construct the survey (AM) in REDCap as well as acquaintances of researchers that were familiar with the subject matter, but would not qualify for the research itself. Knowledge score was calculated as the number of correct answers provided to the knowledge questions, with a total possible score of 49. Subcategories of knowledge score were calculated for tick-related knowledge (out of 32 points) and disease-related knowledge (out of 17 points).

### 2.2 Participant recruitment

A list of veterinary clinics in Illinois of unknown date and provenance was used to initialize a potential participant recruitment list. Since many of these clinics were found to be permanently closed, a Google search for “[county] AND vet clinic” was employed for all potential counties and all identified clinics were added to the list. All clinics from the combined list located in Central or Southern Illinois were contacted. The boundary line delineating regions of Illinois was established based on the Illinois climate divisions program for congruity of data comparison [28].

Clinics were contacted via phone by a researcher. An email address was acquired on the phone if one had not been previously identified; if an email address was on file, it was confirmed. An email containing a recruitment infographic (see Supplement), a brief paragraph explaining the survey and the research, and a thank you note was sent to each veterinary practice email address. An embedded text link to the survey was included in each email to ensure that if participants clicked on the text link or on the recruitment infographic, they would be directed to the online survey. If participants indicated during the phone contact that they were not comfortable taking an electronic survey, their physical address was recorded, and a paper copy of the survey and consent form was mailed to them. All recruitment was conducted between May and September 2020, and the survey was closed in February 2021. All participants were offered an informative poster on the ticks of Illinois as an incentive for participation after completing the survey. This project was reviewed by the University of Illinois Institutional Review Board and determined to be exempt (protocol #21064). All participants provided e-consent prior to completing the survey.

### 2.3 Statistical analysis

Reported clinic case counts for Lyme disease, anaplasmosis, and ehrlichiosis were modeled using Poisson regression. Total reported case counts, summed across all diseases, were modeled using zero-inflated negative binomial models due to overdispersion. Knowledge scores, both overall and by subcategory, were modeled using Poisson regression.

Covariates considered for analysis involving the number of years in practice or the number of years since receiving tick training were modeled as ordinal variables using an additive approach, while number of tick species of concern was modeled as a continuous integer variable. Practices modeled against knowledge scores included the grouped number of tests for TBDs performed in the last 2 years, when testing for TBD was used, whether they routinely asked clients about exposure to ticks, whether they ask about travel history when a TBD is suspected, whether they routinely provide clients with information about TBDs, and whether they reported diagnosing any test-confirmed cases of TBDs in the last 2 years. Models were fit to all data, and separately to a subset of data assessing only the responses of veterinarians. All models were fit in R version 4.1.2 [29] using the MASS [30] and pscl [31] packages.

Descriptive statistics (counts and proportions) were used to describe attitudes and practices items.

## 3 Results

**Fig. 1:**
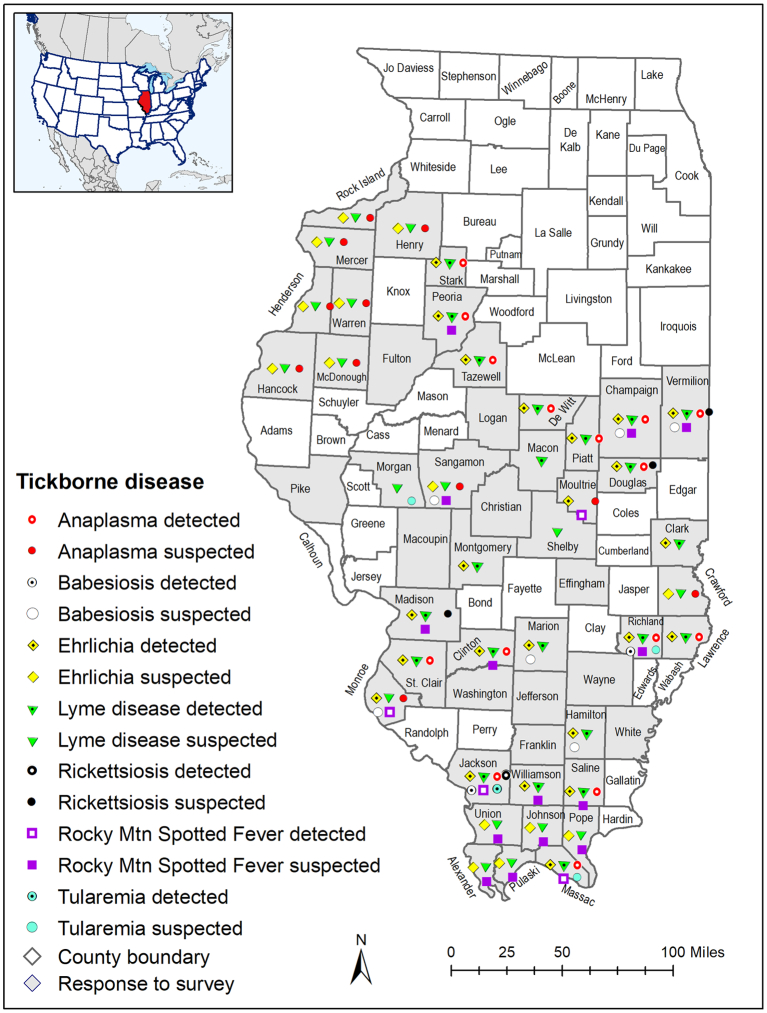
Map of counties in the practice area of participants in the Knowledge, Attitudes, and Practices surveys, indicating in which counties tickborne diseases had been diagnosed or suspected. Detected indicates that a veterinary professional reported diagnosing the disease in an animal and only reported practicing in one county. Suspected included two categories: the veterinary professional indicated that a disease was present but not that they had diagnosed the disease, or the veterinary professional indicated that they had diagnosed the disease but reported a multi-county practice area.

A total of 205 veterinary clinics in Illinois were contacted by mail (*n* = 8) or email (*n* = 197), with reminders sent to 161 clinics. In response, 72 individuals consented to participate and completed at least part of the survey. A description of participants is shown in Table 1, and a map of their practice areas is shown in Fig. 1.

**Table 1:** Demographics of survey participants.

Question	Answer	Number (%)
n		72
Age	<18	3 (4.2)
	18–24	4 (5.6)
	25–34	15 (20.8)
	35–44	14 (19.4)
	45–54	13 (18.1)
	55–64	14 (19.4)
	65+	5 (6.9)
	No answer	4 (5.6)
Gender	Female	43 (59.7)
	Male	23 (31.9)
	Nonbinary	1 (1.4)
	No answer	5 (6.9)
Practice type	Small animal only	47 (65.3)
	Small animal predominant	15 (20.8)
	Mixed animal	8 (11.1)
	Large animal predominant	1 (1.4)
	No answer	1 (1.4)
Position	Veterinary staff member	21 (29.2)
	Licensed veterinary technician	7 (9.7)
	Veterinarian	44 (61.1)
How many years have you been in practice?	<5	5 (6.9)
	6–10	13 (18.1)
	11–15	15 (20.8)
	16–20	1 (1.4)
	21–25	8 (11.1)
	26–30	11 (15.3)
	>30	17 (23.6)
	No answer	2 (2.8)
How many years have you been practicing in your current area?	<5	10 (13.9)
	6–10	12 (16.7)
	11–15	14 (19.4)
	16–20	6 (8.3)
	21–25	6 (8.3)
	26–30	12 (16.7)
	>30	9 (12.5)
	No answer	3 (4.2)
Have you ever been trained on tick species or tick-borne diseases?	No	25 (34.7)
	Yes	46 (63.9)
	No answer	1 (1.4)
How long ago was your training in years?	<5	30 (41.7)
	5–10	7 (9.7)
	>10	7 (9.7)
	No training	28 (38.9)

### 3.1 Knowledge

There was a total of 49 points available for the knowledge scores, 32 related to ticks and 17 related to tick-borne disease (Supplemental Table 1). When considering the knowledge of veterinary professionals, it was found that very few questions (5/49) were answered correctly by more than 75% of respondents. In comparison a large number (23/49) were answered correctly by fewer than 50% of respondents. The range of overall scores was between 8 and 36, with tick-related scores ranging from 6 to 25 and disease-related scores ranging from 2 to 12. Individuals tended to have similar sub-scores, with a correlation of 0.92 (95% CI: 0.88, 0.95).

**Fig. 2:**
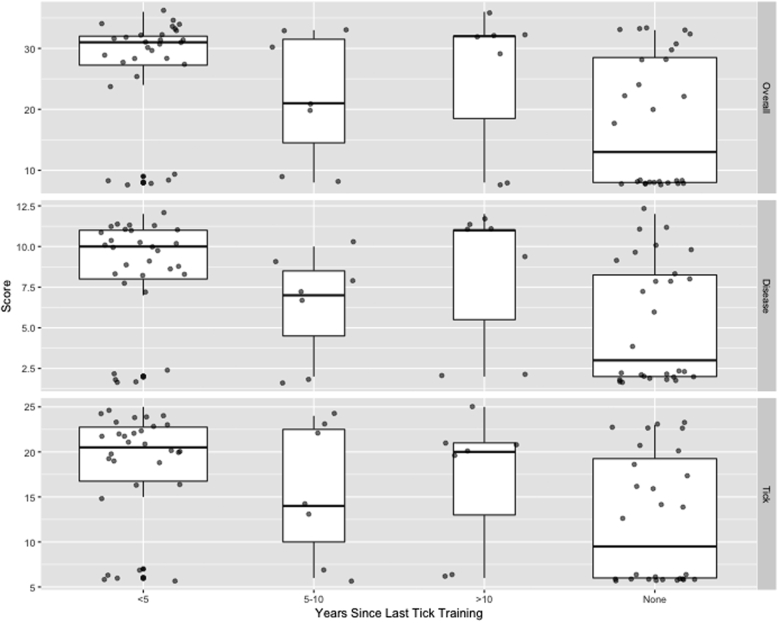
Knowledge scores of veterinary professionals in Illinois regarding ticks, tick-borne diseases, and both ticks and tick-borne diseases (overall). Thick lines indicate median, boxes indicate interquartile range (IQR), and whiskers indicate 1.5*IQR; box width is proportional to number of respondents represented. Points are individual scores.

For knowledge of TBDs, only the category of any training (compared to no training) was significantly associated with score; those without any training were predicted to have a below-average knowledge score of 5.4/17 (95% CI: 4.6, 6.3), while those with training in the last 5 years were predicted to have an above-average disease knowledge score of 8.5 (95% CI: 7.5, 9.6) (Table 2). For knowledge of ticks and overall knowledge score, having been in practice for at least 10 years and having had some TBD training were positively associated with scores (Table 2). However, those whose training was more than 5 years ago had a significantly lower score than those whose training was more recent (Table 2). The same effect is not seen in those whose training was more than 10 years ago, but there were only 7 respondents in that category. The predicted overall score for those with less than 10 years in practice was below average at 20.7/49 (95% CI: 18.8, 22.8), while the predicted score was 24.8 (95% CI: 22.4, 27.3) for those with 10–20 years in practice and 23.1 (95% CI: 21.6, 24.7) for those with more than 20 years in practice (Fig. 2). The predicted overall score for those with no training was 17.8 (95% CI: 16.3, 19.5), while that for those with training in the last 5 years was 27.1 (95% CI: 25.3, 29.0) (Fig. 2).

**Table 2:** Results of Poisson models for scores related to overall knowledge, tick knowledge, and tick-borne disease knowledge based on survey responses from 72 veterinary professionals in Illinois. Results in italics were significant at the *p* < 0.1 level.

		Disease Knowledge		Tick Knowledge		Total Knowledge	
		Training	Practice	Training	Practice	Training	Practice
Intercept		*1.68*	*1.81*	*2.52*	*2.68*	*2.88*	*3.03*
Years in Practice	>10		0.20		*0.17*		*0.18*
	>20		−0.002		−0.10		−0.07
Years Since Tick Training	>0	*0.46*		*0.40*		*0.42*	
	>5	−0.28		*−0.18*		*−0.21*	
	>10	0.25		0.09		0.14	
AICc		413.87	430.57	560.05	589.33	716.34	764.90

The only practice significantly associated with overall knowledge score was whether any test-confirmed cases of TBDs had been diagnosed in the last 2 years. Those who had diagnosed cases had a predicted overall score of 29.8 (95% CI: 28.2, 31.6), while those who had not diagnosed cases had a predicted overall score of 14.0 (95% CI: 12.8, 15.4).

### 3.2 Attitudes

When asked to indicate “which ticks you believe are a concern,” 25 participants selected none of the options. *I. scapularis* was most often selected (41/72), followed by *D. variabilis* (40/72), *A. americanum* (37/72), and *R. sanguineus* (38/72). All other ticks listed (*A. maculatum,* 2; *Dermacentor albipictus*, 8; Other, 2) were rarely of concern; one participant wrote in “Probable Longhorn” for another tick of concern, and the other participant who selected Other did not provide further information. Of those participants listing ticks of concern, the majority were concerned about 3 or 4 different species (13 and 15 respondents, respectively); only one participant selected all 6 listed species, and none selected all 6 plus the “Other” option.

In question six of the Attitudes section of the survey, respondents were asked if they would like to “receive training on ticks and tick-borne diseases” as a simple yes or no multiple choice question. To follow up, question seven in the survey was “What tick and tick-borne disease topics would you like further training in? (Please select all that apply. If you selected ‘No’ in question number 6 above, please skip this question.)” Answer choices to be selected included tick species, tick removal, tick-borne diseases in your area, testing for tickborne diseases, treatment of tick-borne disease, community outreach/communication about ticks and diseases, and other (please specify:_____). Respondents who responded “yes” and reported wanting training in tick species and tick removal had significantly higher knowledge scores related to ticks, and those who reported wanting training in TBDs in their practice area, testing for TBDs, or treatment of TBDs had significantly higher knowledge scores related to TBDs (*p* < 0.001 for all comparisons) (Table 3). Although one person suggested they would like training in another topic, they did not provide further detail when asked what that topic would be. To continue determining where veterinary professionals feel they would gain useful knowledge on ticks and tick-borne diseases, respondents were asked in question nine in the attitudes section from where they would like to receive further outreach. Illinois Department of Public Health (IDPH), the American Veterinary Medical Association (AVMA), the University of Illinois, or Other (Please specify: _____).Of the 41 participants who would like more outreach on ticks and TBDs, 10 said they would like outreach from all three suggested sources, 11 said they would like outreach from 2 of the 3 suggested sources, and 19 selected only one of the suggested sources. The university was the most commonly selected (27/41), while IDPH and AVMA were each selected 19 times and “Other” was not selected. One participant said they would like more outreach but did not select a preferred source.

**Table 3:** Results of zero-inflated negative binomial models for total estimated cases of tick-borne diseases diagnosed in the last two years by veterinary professionals in Illinois. Results in italics were significant at the p < 0.1 level.

		Any Training		Number of Ticks of Concern		Test Decision		Any Disease Concern		Years Since Training		Years in Practice		Combined	
		Count	Logit	Count	Logit	Count	Logit	Count	Logit	Count	Logit	Count	Logit	Count	Logit
Intercept		*2.40*	*−3.52*	1.16	1.74	3.96	−8.88	−0.92	−9.12	*3.28*	0.16	*3.40*	−0.45	−0.83	−36.1
Any tick training		1.17	−6.04												
Number of tick species of concern				*0.63*	−3.66									0.24	6.3
When to Test (routine is base)	TBD suspect					*−1.22*	5.85							*−1.26*	−2.05
	Rule out TBD					*−1.58*	5.34							−1.21	21.11
Tick-borne disease is a concern								*4.40*	−2.92					*3.95*	−11.39
Years since tick training (none is base)	Any training									0.25	−2.67				
	>10									−1.34	−1.45				
	>5									−0.54	1.36				
Years in practice (<10 is base)	>10											−0.38	−3.10		
	>20											0.28	2.39		
AICc		256.89		269.70				396.35		458.01		462.27		385.6	

### 3.3 Practices

Of the 72 respondents, 32 reported no test-confirmed cases of TBD in their practice in the prior 2 years, while 15 reported fewer than 10 cases and a further 18 reported fewer than 50 cases. One participant estimated 300 test-confirmed cases of TBD in the prior 2 years, split evenly among Lyme disease, anaplasmosis, and ehrlichiosis. There were no reported test-confirmed cases of *Borrelia miyamotoi*, Bourbon virus, Heartland virus, Powassan virus, or STAR-I, and only one case each of rickettsiosis or tularemia and two of babesiosis. Ehrlichiosis was the most commonly diagnosed, with 702 cases across 33 respondents. Lyme disease (449 cases from 33 respondents) and anaplasmosis (265 cases from 23 respondents) were also common.

Overall, 30.6% of respondents reported testing when TBDs were suspected, and only 27.8% of respondents reported testing routinely. Of these 42 respondents who reported when they tested, 37 (88%) also reported TBD to be a concern in their area, compared to only 3 of the 26 respondents (11%) who did not give a response to the question of when they tested. Furthermore, 13/23 respondents who reported that clients decline testing due to cost, were primarily recommending testing when tick-borne diseases were suspected (Supplemental Table 3). The mostcommonly mentioned test used was the IDEXX 4DX, which was listed as preferred by 37 of the 45 who answered the question. Most respondants mentioned the speed and convenience of the test; some specified that they would confirm with PCR. Only four respondants mentioned sending samples to an external lab without screening first.

As the majority of respondents (82%) reported that they rely on a test that screens for Lyme, ehrlichiosis, and anaplasmosis only, it is to be expected that these were the only three diseases diagnosed by more than 10% of participants (Supplemental Table 4). The estimated number of cases diagnosed were highly correlated among these three diseases (pairwise correlations between 0.39 and 0.64). A small number of participants also reported diagnosing Rocky Mountain spotted fever, but two of the seven did not estimate the number of cases, and the remaining estimated diagnosing fewer than five cases over two years. Given the low case numbers for individual diseases, all models for factors associated with tick-borne disease diagnosis were conducted over the sum of all case number estimates.

Only three variables (i.e. the number of tick species listed as a concern, when they test for tick-borne disease, and whether tick-borne diseases were considered a concern), were significantly associated with the number of cases, and only for individuals reporting any cases diagnosed (Table 3). Increased concern and routine testing were associated with a higher number of TBDs diagnosed if any TBDs were diagnosed. As these three variables were found not to be significantly correlated, a multivariable model was built using all three. This found that the coefficients were stable when combined, although the number of tick species of concern and testing to rule out TBD were no longer significant.

## 4 Discussion

Our results of a survey of veterinary professionals in Central and Southern Illinois demonstrated that while many veterinarians are familiar with ticks and tick-borne diseases in this area, their amount of knowledge varies considerably, as does the number of cases diagnosed.

Only a small percentage of the questions within the Knowledge section of the survey were answered correctly (only 5/49 were answered correctly by greater than 75% of respondents). This result indicates a lack of knowledge related to many aspects of ticks and tick-borne diseases in the population surveyed. This can be explained in part by our finding that recent training was significantly associated with higher knowledge scores; very little veterinary-focused tick training has been made available in this area in recent years. Research supports that targeted tick-borne disease training can significantly increase knowledge scores among local public health department employees [32]. Based on the surveyed topics that received lower correct response rates, we suggest that training for veterinary professionals should focus on the specific tick species present in the practice area, where ticks typically attach to a host body (both animal and human), the diagnostics available for vector-borne diseases, and potentially alpha-gal sensitivity. Although domestic animals are unlikely to be affected by alpha-gal allergies, it has clinical implications for humans. Veterinarians play a substantial role in communicating the public health risks of TBDs, so it may be important for them to be aware of this growing problem.

Lyme disease, anaplasmosis, and ehrlichiosis were the most commonly reported tick-borne disease cases diagnosed by the study participants. It is no coincidence that these are the three tick-borne diseases included in the IDEXX Snap 4DX test [33], which was the preferred test for 82% of practitioners reporting a preferred test. While this set of three diseases also represents the most commonly diagnosed human TBDs in Illinois [14,34], there is a risk of underdiagnosing less common TBDs not included in this panel in veterinary patients due to diagnostic bias. It is also important to note that 32% of participants cited cost as the main reason why clients decline TBD testing, limiting the use of more comprehensive diagnostics.

In assessing clinical rationale and timing of tick-borne disease testing, we observed that a concern for tick-borne disease correlated with an increased knowledge score, so there is the potential that this attitude may also correlate to an increased testing rate. While the relationship is correlative only, this is supported by the analysis of practices by knowledge score, which showed that participants reporting diagnosed cases had significantly higher scores. Thus, any surveillance program based on reports of diagnosed TBD is likely to experience ascertainment bias in areas where TBDs are newly emerging or not known to be established. The veterinarians practicing in these areas are unlikely to test for diseases they do not believe are a local concern.

Therefore, promoting awareness and increasing the likelihood that veterinarians will test and recognize TBDs may require partnerships with researchers [21] and industry. These partnerships may contribute to developing diagnostic tests and a national TBD veterinary surveillance system that can identify and map the distribution and occurrence of TBD in animals and the shared TBD in domestic animals and humans.

Of respondents who reported testing only when suspecting TBDs, a majority also reported that the primary reason for clients to decline testing was cost. This suggests that those animals may be treated pre-emptively, and that those potential TBDs would go officially undiagnosed due to financial limitations. This creates another bias, in that TBDs would therefore be less likely to be reported in low-income areas. As public health responses to TBDs are often based on the location of reported cases, including veterinary case data, this could lead to disparities in control and education efforts. It could also create disparities in veterinary care, as awareness of locally prevalent TBDs was found here to be an important factor in client education; those who reported believing that client education on TBDs was important had higher knowledge scores than those who did not respond.

Some questions in this veterinary KAP survey were notably targeted towards humans and not animals because the CDC's resource pages for ticks and TBDs were used to create the questionnaire. The CDC is presumed to be the leading quick reference for information on tick-borne diseases for the average person. It is visually appealing and digestible while containing information on tick environments, feeding habits, and prevention.

Animal-specific resources on ticks and tick-borne diseases are fewer in number. As a result, they tend to focus more on treating TBDs and less on factors that could be leveraged to prevent them. Furthermore, there is no national systematic and routine surveillance of ticks of animal health importance capturing the information from practicing veterinarians beyond the CAPC system [24]. The lack of a national TBD resource for veterinarians prevents the veterinary community from having reliable, widely available information that shows the geographic distributions of ticks and veterinary TBD cases. Beyond the benefit to the veterinary community, a veterinary national TBD resource may help improve our understanding of changing risks of human exposure to ticks and TBD from pets and domestic animals sharing spaces with humans.

In recent years, TBDs are gaining attention from the public [35], medical [36], and veterinary professionals [37]. This increased focus is not surprising since people and companion animals are vulnerable to many of the same TBDs, and human cases of TBDs have risen dramatically in the United States over the past decade [1]. Although not measured in this study, it is not unrealistic to recommend that more collaboration between human and animal medicine would be beneficial [21,23] and that both medical fields strive towards a common goal of population health and treatment and prevention of TBDs.

Training to improve TBD knowledge could be in the form of veterinary continuing education on ticks and TBDs. In addition, building capacity for training veterinarians in parasitology using partnerships between academia and industry may strengthen the resources, knowledge, and understanding of ticks and tick-borne pathogens in the veterinary community. Perhaps it is also worth considering training that brings together participants from the veterinary and human medical fields. A One Health approach to the training may contribute to building networks between the medical fields to address the challenges surrounding diagnosis and testing, and build communication pathways between medical practitioners and veterinarians. Such an approach would enhance the role veterinarians play in public health [38], which is beneficial for both the profession and for public health as a whole.

This study is limited by the potential for response bias and by the limited range of the study area. It is possible that our sample was weighted towards those with an interest in ticks and TBDs, which could bias our results towards higher interest and knowledge scores. However, given the low knowledge scores of many respondents, we believe this bias to be small. More importantly, our sample was limited to veterinary professionals in Central and Southern Illinois. This area is on the leading edge of the *I. scapularis* range expansion and is not known to be a high-risk area for Lyme disease, the most common and most publicized TBD. As such, the results may not be applicable in areas with more established TBD risk, such as the Northeast or Upper Midwest, US. The area is also primarily rural farmland, and therefore the results may not be applicable in more suburban or urban areas. We would recommend future research to expand this survey nationally, so that regional differences can be explored.

The following are the supplementary data related to this article.

**Supplementary Fig. 1:**
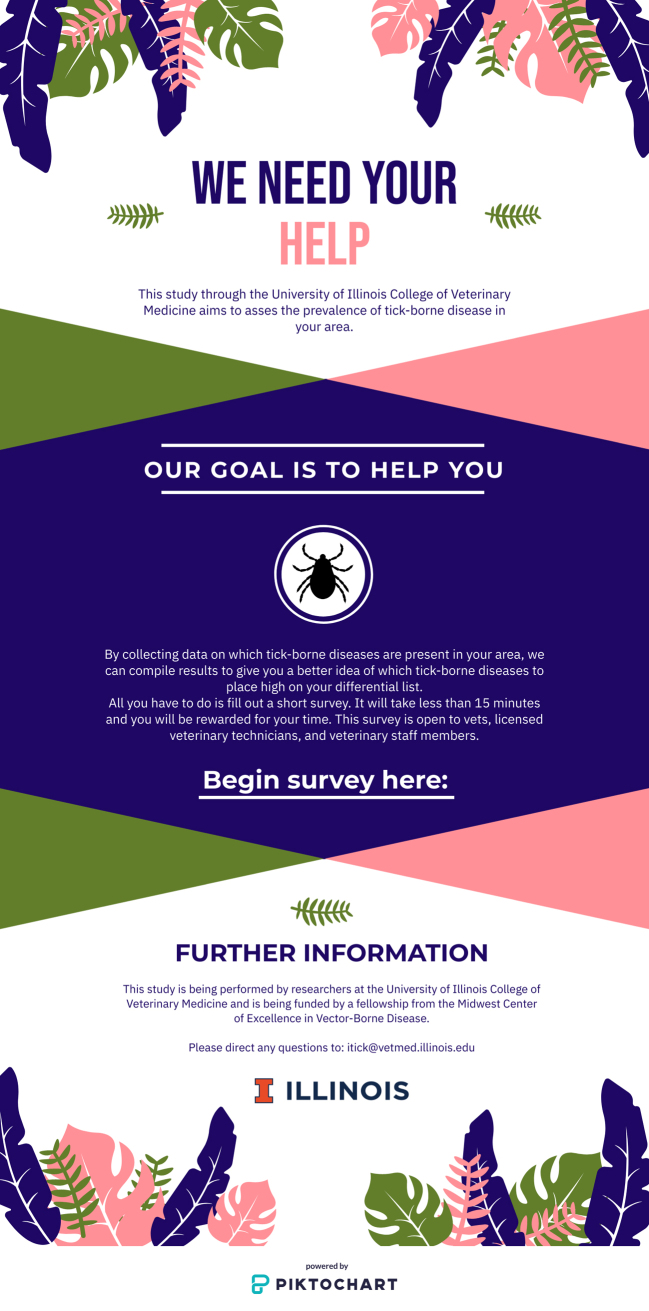
Poster used to recruit survey participants

Supplementary material 1 Distribution of responses to survey by question. Supplementary material 1

Supplementary material 2 Text of survey as downloaded from REDCap Supplementary material 2

## CRediT authorship contribution statement

**Samantha D. Crist:** Conceptualization, Methodology, Investigation. **Heather Kopsco:** Conceptualization, Methodology. **Alexandria Miller:** Methodology, Investigation. **Peg Gronemeyer:** Formal analysis, Data curation, Writing – review & editing, Visualization. **Nohra Mateus-Pinilla:** Conceptualization, Methodology. **Rebecca L. Smith:** Conceptualization, Methodology, Formal analysis, Data curation, Visualization.

## Declaration of Competing Interest

The authors declare that there were no conflicts of interest.
