# Supplementary material for: Knowledge, attitudes, and practices of veterinary professionals towards ticks and tick-borne diseases in Illinois
Source: One Health. 2022 Apr 24;14:100391. doi: 10.1016/j.onehlt.2022.100391 (PMC9171534; doi:10.1016/j.onehlt.2022.100391)
Supplement: Supplementary material 1 — Distribution of responses to survey by question. [file mmc2.pdf]

Veterinary KAP Survey PID 373

Project Home | Project Setup | Online Designer | Data Dictionary | Codebook

Codebook

Data Dictionary Codebook

04-26-2021 9:37pm

Collapse all instruments

#	Variable / Field Name	Field Label<br>*Field Note*	Field Attributes (Field Type, Validation, Choices, Calculations, etc.)
Instrument: **Veterinary KAP Survey** (veterinary_kap_survey) Enabled as survey			Collapse
1	participant_id	Participant ID	text
2	consent_form	Knowledge, Attitudes, and Practices of Veterinary Professionals Towards Ticks and Tick-Borne Diseases SurveyYou are being asked to participate in a voluntary research study. The purpose of this study is to assess your knowledge and experience with ticks and tick-borne diseases. Risks related to this research are not beyond what you would already experience in your everyday life. The researchers will not have access to your individually identifiable information. The benefits related to this research include improving our ability to design outreach and educational materials for veterinary professionals in Illinois. Principal Investigator Name and Title: Rebecca Smith, Associate Professor of EpidemiologyDepartment and Institution: Department of Pathobiology, University of Illinois College of Veterinary MedicineContact Information: 217-300-1428 (phone); rlsdvm@illinois.edu (email)What procedures are involved? The study procedures include completing an online survey. This research will be collected using REDCap, which is a data collection and management application. You will need to participate one time. Your participation will last approximately 15 minutes.Will my study-related information be kept confidential?Faculty, students, and staff will not have access to	descriptive

		any of your individually identifiable information. Confidentiality will be maintained to the extent of laws and university policies. Personal identifiers will not be collected, published, or presented.Will I be reimbursed for any expenses or paid for my participation in this research?You will be given the option to receive a poster on ticks and tick-borne diseases in Illinois, suitable for hanging in a clinic. Can I withdraw or be removed from the study?If you decide to participate, you are free to withdraw your consent and discontinue participation at any time, please just close your browser. Your participation in this research is voluntary. Your decision whether or not to participate, or to withdraw after beginning participation, will not affect your current or future dealings with the University of Illinois at Urbana-Champaign. Will data collected from me be used for any other research?Your de-identified information could be used for future research without additional informed consent.Whom should I contact if I have questions?If you have questions about this project, you may contact the principal investigator, Rebecca Smith, Associate Professor via phone at 217-300-1428 or via email to rlsdvm@illinois.edu; or Nohra Mateus-Pinilla, Director, Wildlife Veterinary Epidemiology Laboratory via phone at 217-333-6856 or via email to nohram@illinois.edu. If you have any questions about your rights as a participant in this study or any concerns or complaints, please contact the University of Illinois at Urbana-Champaign Office for the Protection of Research Subjects at 217-333-2670 or via email at irb@illinois.edu.Please print this consent form if you would like to retain a copy for your records.I have read and understand the above consent form. I certify that I am 18 years old or older. By clicking the "Submit" button to enter the survey, I indicate my willingness to voluntarily take part in this study.	

3	introduction	Section Header: *Start*<br>Greetings all. Thank you for participating in this short survey. Participation is voluntary and declining to participate or finish the survey will involve no penalties. The goal of this survey is to assess the knowledge, attitudes, and practices among veterinarians and veterinary technicians in regards to tick-borne diseases. Participation should only take about 15 minutes of your time. Information will remain confidential and identifying information will be collected in a categorical fashion to protect identities of participants. No foreseeable risks are associated with the taking of this survey. Benefits include an informational poster to express our gratitude for your participation and the furthering of science related to public health in your area. Please send any questions to: itick@vetmed.illinois.edu.<br><br>We greatly appreciate your participation. As thanks, upon completion you will be mailed a poster for your waiting room to show your support of important public health research.	descriptive
4	age	Section Header: *Demographics*<br>Please select the group that includes your age.	dropdown<br>1 Under 18<br>2 18-24<br>3 25-34<br>4 35-44<br>5 45-54<br>6 55-64<br>7 65 and above
5	gender	Please select the gender you identify with most.	radio<br>1 Female<br>2 Male<br>3 Nonbinary<br>4 Other/Prefer not to say
6	county	Please list the county/counties in which you practice.	text, Required

7	practice_distribution	Please select your clinic's practice distribution.	radio<br>1 Small animal only<br>2 Small animal predominant<br>3 Mixed animal<br>4 Large animal predominant<br>5 Large animal only<br>6 Production animal only
8	species_specific<br>Show the field ONLY if:<br>[practice_distribution] = '1' or<br>[practice_distribution] = '2' or<br>[practice_distribution] = '4' or<br>[practice_distribution] = '3' or<br>[practice_distribution] = '5' or<br>[practice_distribution] = '6'	If your clinic is single species exclusive, please identify it here. (Ex. Swine only).	text
9	job_position	Please indicate if you are a veterinarian, licensed veterinary technician, or veterinary staff member.	radio, Required<br>2 Veterinarian<br>1 Licensed veterinary technician<br>0 Veterinary staff member
10	years_in_practice	How many years have you been in practice?	dropdown<br>1 Less than 5 years<br>2 6-10 years<br>3 11-15 years<br>4 16-20 years<br>5 21-25 years<br>6 26-30 years<br>7 Over 30 years

11	years_in_area	How many years have you been practicing in your current area?	dropdown<br>1 Less than 5 years<br>2 6-10 years<br>3 11-15 years<br>4 16-20 years<br>5 21-25 years<br>6 26-30 years<br>7 Over 30 years
12	training	Have you ever been trained on tick species or tick-borne diseases?	yesno<br>1 Yes<br>0 No
13	training_years_ago<br>Show the field ONLY if:<br>[training] = '1'	How long ago was your training in years?	text
14	kind_of_training<br>Show the field ONLY if:<br>[training] = '1'	What kind of training did you receive?	notes
15	tick_species_near	Section Header: *Knowledge In this section, we would like to gain knowledge of your current understanding of ticks and tick-borne diseases. We are simply interested in what you know.*<br>Please list any tick species that you know of that are near you.	notes
16	disease_species	Please indicate any species of tick you recorded above that can carry disease.	notes
17	time_to_transmit	How long does a tick have to be attached to you or an animal to transmit disease?	radio<br>0 Seconds<br>3 Minutes<br>2 Hours<br>1 Days

<table>
<tr><td>18</td><td>bite</td><td>Can you always tell if a tick has bitten you?</td><td>yesno<table><tr><td>1</td><td>Yes</td></tr><tr><td>0</td><td>No</td></tr></table></td></tr>
<tr><td>19</td><td>tick_habitat</td><td>Which of the below are considered preferred habitats for ticks? (Please select as many as you believe apply).</td><td>checkbox<table><tr><td>0</td><td>tick_habitat___0</td><td>Desert</td></tr><tr><td>3</td><td>tick_habitat___3</td><td>Grasslands</td></tr><tr><td>4</td><td>tick_habitat___4</td><td>Woodlands</td></tr><tr><td>1</td><td>tick_habitat___1</td><td>Swamps</td></tr><tr><td>2</td><td>tick_habitat___2</td><td>Agricultural fields</td></tr></table></td></tr>
<tr><td>20</td><td>bite_locations</td><td>Where on the body are locations where ticks commonly attach and bite? (Please select all that apply).</td><td>checkbox<table><tr><td>0</td><td>bite_locations___0</td><td>Under the arms</td></tr><tr><td>1</td><td>bite_locations___1</td><td>In hair</td></tr><tr><td>2</td><td>bite_locations___2</td><td>Behind the ears</td></tr><tr><td>3</td><td>bite_locations___3</td><td>Inside the belly button</td></tr><tr><td>4</td><td>bite_locations___4</td><td>Wrists</td></tr><tr><td>5</td><td>bite_locations___5</td><td>Back of the knees</td></tr><tr><td>6</td><td>bite_locations___6</td><td>Between the legs</td></tr><tr><td>7</td><td>bite_locations___7</td><td>Shoulders</td></tr><tr><td>8</td><td>bite_locations___8</td><td>Around the waist</td></tr></table></td></tr>
<tr><td>21</td><td>diseases_present</td><td>Please list any tick-borne diseases that are present in your area. If you feel there are none, please type N/A.</td><td>notes</td></tr>
</table>

22	test_positive_diseases	Please indicate tick-borne diseases for which you have had patients test positive in the past two years. In the following questions please estimate the number of cases that are test confirmed that you have seen. Please limit your response to those cases occurring in the past two years. (Please select all that apply and take your best guess if you cannot recall exactly how many cases of each disease have appeared in your clinic).	checkbox, Required<br>0 | test_positive_diseases___0 | Anaplasmosis<br>1 | test_positive_diseases___1 | Babesiosis<br>2 | test_positive_diseases___2 | Borrelia miyamotoi<br>3 | test_positive_diseases___3 | Lyme disease<br>4 | test_positive_diseases___4 | Bourbon virus<br>5 | test_positive_diseases___5 | Ehrlichiosis<br>6 | test_positive_diseases___6 | Heartland virus<br>7 | test_positive_diseases___7 | Powassan disease<br>8 | test_positive_diseases___8 | Rickettsiosis<br>9 | test_positive_diseases___9 | Rocky Mountain Spotted Fever<br>10 | test_positive_diseases___10 | Southern Tick Associated Rash Illness (STARI)<br>11 | test_positive_diseases___11 | Tularemia
23	anaplasmosis<br><br>Show the field ONLY if: [test_positive_diseases(0)] = '1'	Cases of anaplasmosis:	text (number)
24	babesiosis<br><br>Show the field ONLY if: [test_positive_diseases(1)] = '1'	Cases of babesiosis:	text (number)
25	borrelia_miyamotoi<br><br>Show the field ONLY if: [test_positive_diseases(2)] = '1'	Cases of Borrelia miyamotoi:	text (number)

26	lyme<br><br>Show the field ONLY if:<br>[test_positive_diseases(3)] = '1'	Cases of Lyme disease:	text (number)
27	bourbon_virus<br><br>Show the field ONLY if:<br>[test_positive_diseases(4)] = '1'	Cases of Bourbon virus:	text (number)
28	ehrlichiosis<br><br>Show the field ONLY if:<br>[test_positive_diseases(5)] = '1'	Cases of ehrlichiosis:	text (number)
29	heartland_virus<br><br>Show the field ONLY if:<br>[test_positive_diseases(6)] = '1'	Cases of Heartland virus:	text (number)
30	powassan_disease<br><br>Show the field ONLY if:<br>[test_positive_diseases(7)] = '1'	Cases of Powassan disease:	text (number)
31	rickettsiosis<br><br>Show the field ONLY if:<br>[test_positive_diseases(8)] = '1'	Cases of rickettsiosis:	text (number)
32	rmsf<br><br>Show the field ONLY if:<br>[test_positive_diseases(9)] = '1'	Cases of Rocky Mountain Spotted Fever:	text (number)
33	stari<br><br>Show the field ONLY if:<br>[test_positive_diseases(10)] = '1'	Cases of Southern Tick Associated Rash Illness (STARI):	text (number)

34	tularemia<br>Show the field ONLY if:<br>[test_positive_diseases(11)] = '1'	Cases of tularemia:	text (number)
35	symptoms	What are symptoms of tick-borne disease? (Please select all that apply).	checkbox<br>1 symptoms___1 Fever<br>2 symptoms___2 Hair loss<br>3 symptoms___3 Joint pain<br>4 symptoms___4 Rash<br>5 symptoms___5 None of the above
36	risk_factors	What are risk factors for tick-borne diseases? (Please select as many as you feel apply).	checkbox<br>1 risk_factors___1 Working outdoors in wooded areas<br>2 risk_factors___2 Being bitten by a tick<br>3 risk_factors___3 Working of a golf course<br>4 risk_factors___4 Having a tall un-mowed grass lawn
37	tests	What tests are available to test for tick-borne diseases?	notes
38	test_preference	Which tests for tick-borne diseases do your prefer and why?	notes
39	samples	What samples should be taken for the tests you mentioned above? (Please select all that apply).	checkbox<br>1 samples___1 Serum<br>2 samples___2 Blood<br>3 samples___3 Joint fluid<br>4 samples___4 Other
40	sample_other<br>Show the field ONLY if:<br>[samples(4)] = '1'	Please specify:	text
41	treatments	What treatments are available for tick-borne diseases?	notes

42	alpha_gal	Have you ever heard of the alpha-gal/red meat allergy?	yesno<br>1 Yes<br>0 No
43	alpha_gal_cause	What causes the alpha-gal/ red meat allergy? (If you are uncertain, please give your best guess).	notes
44	alpha_gal_test	Is there a test for the alpha-gal/red meat allergy?	yesno<br>1 Yes<br>0 No
45	alpha_gal_treatment	How do you treat the alpha-gal/red meat allergy?	radio<br>1 Antibiotics<br>2 Monoclonal antibody therapy<br>3 Antigen stimulus therapy<br>4 There is no therapy
46	tick_concern	Section Header: *Attitudes In this section, we would like to assess your opinions and how you feel about ticks and tick-borne diseases.*<br>Do you believe that ticks are a concern in your area?	yesno<br>1 Yes<br>0 No

47	ticks_of_concern	Please indicate which ticks you believe are a concern. (Please select all that apply).	checkbox<br>1 ticks_of_concern___1 The Blacklegged tick or Deer Tick (Ixodes scapularis)<br>2 ticks_of_concern___2 The Brown Dog tick (Rhipicephalus sanguineus)<br>3 ticks_of_concern___3 The Gulf Coast tick (Amblyomma maculatum)<br>4 ticks_of_concern___4 The Lone Star tick (Amblyomma americanum)<br>5 ticks_of_concern___5 The American Dog tick (Dermacentor variabilis)<br>6 ticks_of_concern___6 The Winter tick (Dermacentor albipictus)<br>7 ticks_of_concern___7 Other(s)<br>8 ticks_of_concern___8 Not applicable
48	tick_other<br>Show the field ONLY if:<br>[ticks_of_concern(7)] = '1'	Please specify:	text
49	education_importance	Do you feel that client education about ticks and tick-borne diseases is important?	yesno<br>1 Yes<br>0 No
50	disease_concern	Do you think that tick-borne diseases are a concern in your area?	yesno<br>1 Yes<br>0 No
51	diseases_of_concern<br>Show the field ONLY if:<br>[disease_concern] = '1'	If you selected "Yes" above, please list the tick-borne diseases you are concerned about in your area.	notes

<table>
<tr><td>52</td><td>training_yn</td><td>Would you like to have training on ticks and tick-borne diseases?</td><td>yesno<table><tr><td>1</td><td>Yes</td></tr><tr><td>0</td><td>No</td></tr></table></td></tr>
<tr><td>53</td><td>training_topics</td><td>What tick and tick-borne disease topics would you like further training in? (Please select all that apply).</td><td>checkbox<table><tr><td>1</td><td>training_topics___1</td><td>Tick species</td></tr><tr><td>2</td><td>training_topics___2</td><td>Tick removal</td></tr><tr><td>3</td><td>training_topics___3</td><td>Tick-borne diseases in your area</td></tr><tr><td>4</td><td>training_topics___4</td><td>Testing for tick-borne diseases</td></tr><tr><td>5</td><td>training_topics___5</td><td>Treatment of tick-borne diseases</td></tr><tr><td>6</td><td>training_topics___6</td><td>Community outreach/communication about ticks and diseases</td></tr><tr><td>7</td><td>training_topics___7</td><td>Other</td></tr></table></td></tr>
<tr><td>54</td><td>training_other
Show the field ONLY if:
[training_topics(7)] = '1'</td><td>Please specify:</td><td>notes</td></tr>
<tr><td>55</td><td>public_outreach</td><td>Do you feel like more public outreach about ticks and tick-borne diseases from the Illinois Department of Public Health (IDPH), American Veterinary Medical Association (AVMA), or the University of Illinois would benefit your area?</td><td>yesno<table><tr><td>1</td><td>Yes</td></tr><tr><td>0</td><td>No</td></tr></table></td></tr>
<tr><td>56</td><td>outreach_agency</td><td>Who would you like to receive public outreach from?</td><td>checkbox<table><tr><td>1</td><td>outreach_agency___1</td><td>Illinois Department of Public Health (IDPH)</td></tr><tr><td>2</td><td>outreach_agency___2</td><td>American Veterinary Medical Association (AVMA)</td></tr><tr><td>3</td><td>outreach_agency___3</td><td>The University of Illinois</td></tr><tr><td>4</td><td>outreach_agency___4</td><td>Other</td></tr></table></td></tr>
</table>

<table>
<tr><td>57</td><td>outreach_other
Show the field ONLY if:
[outreach_agency(4)] = '1'</td><td>Please specify:</td><td>text</td></tr>
<tr><td>58</td><td>look_for_ticks</td><td>Section Header: Practices For this section, we would like to gain knowledge of your current practices regarding ticks and tick-borne diseases.
Do you routinely look for ticks on yourself and/or patients?</td><td>yesno
<table><tr><td>1</td><td>Yes</td></tr><tr><td>0</td><td>No</td></tr></table></td></tr>
<tr><td>59</td><td>ticks_removed</td><td>If you have removed ticks from yourself and/or your patients, what species of tick have you found? (Please select as many as apply).</td><td>checkbox
<table>
<tr><td>1</td><td>ticks_removed___1</td><td>The Blacklegged tick or Deer Tick (Ixodes scapularis)</td></tr>
<tr><td>2</td><td>ticks_removed___2</td><td>The Brown Dog tick (Rhipicephalus sanguineus)</td></tr>
<tr><td>3</td><td>ticks_removed___3</td><td>The Gulf Coast tick (Amblyomma maculatum)</td></tr>
<tr><td>4</td><td>ticks_removed___4</td><td>The Lone Star tick (Amblyomma americanum)</td></tr>
<tr><td>5</td><td>ticks_removed___5</td><td>The American Dog tick (Dermacentor variabilis)</td></tr>
<tr><td>6</td><td>ticks_removed___6</td><td>The Winter tick (Dermacentor albipictus)</td></tr>
<tr><td>7</td><td>ticks_removed___7</td><td>Others</td></tr>
<tr><td>8</td><td>ticks_removed___8</td><td>I don't know/don't remember</td></tr>
</table></td></tr>
<tr><td>60</td><td>other_removed
Show the field ONLY if:
[ticks_removed(7)] = '1'</td><td>Please specify:</td><td>notes</td></tr>
<tr><td>61</td><td>exposure</td><td>Do you routinely ask about tick exposure when talking to clients?</td><td>yesno
<table><tr><td>1</td><td>Yes</td></tr><tr><td>0</td><td>No</td></tr></table></td></tr>
</table>

62	when_to_test	When do you test for tick-borne diseases?	radio<br>1 Routinely<br>2 When suspecting a tick-borne disease<br>3 To rule out a tick-borne disease
63	disease_and_test	Which tick-borne diseases do you test for and what tests do you use?	notes
64	preferred_sample	What samples do you prefer to use/submit? (Please select those that apply).	checkbox<br>1 preferred_sample___1 Serum<br>2 preferred_sample___2 Blood<br>3 preferred_sample___3 Joint fluid<br>4 preferred_sample___4 Other
65	preferred_sample_other<br>Show the field ONLY if:<br>[preferred_sample(4)] = '1'	Please specify:	checkbox
66	test_number	How many tests for tick-borne diseases have you performed in the past year?	radio<br>1 1-10<br>2 10-50<br>3 50-100<br>4 100-200<br>5 200+
67	decline_testing	What is the main reason clients decline testing for tick-borne diseases?	radio<br>1 They find it unnecessary<br>2 Cost is a limiting factor<br>3 Tick-borne disease is not a main differential<br>4 Other
68	decline_other<br>Show the field ONLY if:<br>[decline_testing] = '4'	Please specify:	notes

69	travel_history	When you have a patient present with signs of a tick-borne disease like a fever of unknown origin, joint pain, and lethargy, do you ask about travel history?	yesno<br>1 Yes<br>0 No
70	suspect_places<br>Show the field ONLY if: [travel_history] = '1'	Please list the places (if any) that would make you suspect tick-borne diseases. (Please be as specific as possible).	notes
71	highest_tick_numbers	From which species do you remove the most ticks?	radio<br>1 Cats<br>2 Dogs<br>3 Cattle<br>4 Horses<br>5 Poultry<br>6 Exotics/pocket pets
72	highest_disease_species	In which species do you diagnose the highest number of tick-borne diseases?	radio<br>1 Cats<br>2 Dogs<br>3 Cattle<br>4 Horses<br>5 Poultry<br>6 Exotics/pocket pets
73	your_treatment	How do you treat tick-borne diseases?	notes
74	resistance	What is your level of concern about antimicrobial resistance in regards to the treatment of tick-borne diseases?	dropdown<br>1 Low<br>2 Medium<br>3 High<br>Custom alignment: RH

75	disease_info	Do you provide information about tick-borne diseases to your clients on a routine basis?	yesno<br>1 Yes<br>0 No<br><br>Custom alignment: RH
76	info_form	If you do provide information to your clients about tick-borne diseases, how do you do this?	checkbox<br>1 info_form___1 Flyers/pamphlets<br>2 info_form___2 Verbally<br>3 info_form___3 Refer to a website<br>4 info_form___4 Other<br><br>Custom alignment: RH
77	info_form_other<br>Show the field ONLY if:<br>[info_form(4)] = '1'	Please specify:	text<br>Custom alignment: RH
78	additional_info	Section Header: *Final Questions*<br>Do you have any information you would like to share? If so, please include it here.	notes<br>Custom alignment: RH
79	final_questions	Please include any questions you have about this survey or its content.	notes<br>Custom alignment: RH
80	address	Section Header: *Thank you for completing this survey. Your answers are highly valued and will help improve our knowledge of ticks and tick-borne disease in your area, so that we in-turn can help you formulate a more accurate tick-borne disease differential list. Thank you again for your help. We could never perform a study like this without people like you. Please fill out the brief form below if you would like to receive your complimentary commemorative poster.*<br>Please type your name and mailing address here if you wish to receive your complimentary commemorative poster. Thank you again.	notes, Identifier<br>Custom alignment: RH
81	veterinary_kap_survey_complete	Section Header: *Form Status*<br>Complete?	dropdown<br>0 Incomplete<br>1 Unverified<br>2 Complete
