# Supplementary material for: Knowledge, attitudes, and practices of veterinary professionals towards ticks and tick-borne diseases in Illinois
Source: One Health. 2022 Apr 24;14:100391. doi: 10.1016/j.onehlt.2022.100391 (PMC9171534; doi:10.1016/j.onehlt.2022.100391)
Supplement: Supplementary material 2 — Text of survey as downloaded from REDCap [file mmc3.docx]

**Supplemental Table 1:** Knowledge questions and responses by participants

| Question | | Incorrect Answer (%) | | Correct Answer (%) | |
| --- | --- | --- | --- | --- | --- |
| Tick questions | | | | | |
| Please list any tick species that you know of that are near you | *Ixodes scapularis* | No | 33 (45.8) | Yes | 39 (54.2) |
|  | *Dermacentor variabilis* | No | 43 (59.7) | Yes | 29 (40.3) |
|  | *Amblyomma americanum* | No | 38 (52.8) | Yes | 34 (47.2) |
|  | *Rhipicephalus sanguineus* | No | 42 (58.3) | Yes | 30 (41.7) |
| Please indicate any species of tick you recorded above that can carry disease | *Ixodes scapularis* | No | 30 (41.7) | Yes | 42 (58.3) |
|  | *Dermacentor variabilis* | No | 39 (54.2) | Yes | 33 (45.8) |
|  | *Amblyomma americanum* | No | 36 (50.0) | Yes | 36 (50.0) |
|  | *Rhipicephalus sanguineus* | No | 38 (52.8) | Yes | 34 (47.2) |
| How long does a tick have to be attached to you or an animal to transmit disease? | | Seconds | 11 (15.3) | Days | 6 (8.3) |
|  |  | Minutes | 9 (12.5) | Hours | 28 (38.9) |
|  |  | No answer | 18 (25.0) |  |  |
| Can you always tell if a tick has bitten you? | | Yes | 2 (2.8) | No | 54 (75.0) |
|  |  | No answer | 16 (22.2) |  |  |
| Which of the following are considered preferred habitats for ticks? | Desert | Yes | 5 (6.9) | No | 67 (93.1) |
|  | Grasslands | No | 24 (33.3) | Yes | 48 (66.7) |
|  | Woodlands | No | 25 (34.7) | Yes | 47 (65.3) |
|  | Swamps | Yes | 22 (30.6) | No | 50 (69.4) |
|  | Agricultural | Yes | 42 (58.3) | No | 30 (41.7) |
| Where on the body are locations where ticks commonly attach and bite? | Under arms | No | 33 (45.8) | Yes | 39 (54.2) |
|  | In hair | No | 30 (41.7) | Yes | 42 (58.3) |
|  | Behind ears | No | 31 (43.1) | Yes | 41 (56.9) |
|  | Inside Belly Button | No | 48 (66.7) | Yes | 24 (33.3) |
|  | Wrists | Yes | 12 (16.7) | No | 60 (83.3) |
|  | Back of Knees | No | 36 (50.0) | Yes | 36 (50.0) |
|  | Between Legs | No | 38 (52.8) | Yes | 32 (44.4) |
|  |  | No answer | 2 (2.8) |  |  |
|  | Shoulders | Yes | 15 (20.8) | No | 54 (75.0) |
|  |  | No answer | 3 (4.2) |  |  |
|  | Around Waist | Yes | 35 (48.6) | No | 35 (48.6) |
|  |  | No answer | 2 (2.8) |  |  |
| Which of the following are risk factors for tick-borne diseases? | Working Outdoors | No | 23 (31.9) | Yes | 49 (68.1) |
|  | Tick Bite | No | 22 (30.6) | Yes | 48 (66.7) |
|  |  | No answer | 2 ( 2.8) |  |  |
|  | Working at a Golf Course | No | 48 (66.7) | Yes | 19 (26.4) |
|  |  | No answer | 5 ( 6.9) |  |  |
|  | Unmowed Lawn | No | 17 (23.6) | Yes | 48 (66.7) |
|  |  | No answer | 7 (9.7) |  |  |
| Disease Questions | | | | |  |
| Which of the following are symptoms of tick-borne diseases? | Fever | No | 23 (31.9) | Yes | 49 (68.1) |
|  | Hair Loss | Yes | 10 (13.9) | No | 62 (86.1) |
|  | Joint Pain | No | 23 (31.9) | Yes | 49 (68.1) |
|  | Rash | No | 36 (50.0) | Yes | 36 (50.0) |
|  | None | Yes | 2 (2.8) | No | 70 (97.2) |
| What tests are available to test for tick-borne diseases? | IDEXX | No | 49 (68.1) | Yes | 23 (31.9) |
|  | PCR | No | 57 (79.2) | Yes | 15 (20.8) |
|  | Titers | No | 57 (79.2) | Yes | 15 (20.8) |
| What samples should be taken for the tests you mentioned  above? | Serum | No | 39 (54.2) | Yes | 33 (45.8) |
|  | Blood | No | 29 (40.3) | Yes | 43 (59.7) |
|  | Joint Fluid | No | 68 (94.4) | Yes | 4 (5.6) |
| What treatments are available for tick-borne diseases? | | Nothing/ supportive care only | 29 (40.3) | Antibiotics/ Antiparasitics | 43 (59.7) |
| Have you ever heard of the alpha-gal/red meat allergy? | | No | 30 (41.7) | Yes | 18 (25.0) |
|  |  | No answer | 24 (33.3) |  |  |
| What causes the alpha-gal/ red meat allergy? | Immune response | No | 58 (80.6) | Yes | 14 (19.4) |
|  | Ticks/tick bite | No | 45 (62.5) | Yes | 27 (37.5) |
| Is there a test for the alpha-gal/red meat allergy? | | No | 16 (22.2) | Yes | 26 (36.1) |
|  |  | No answer | 30 (41.7) |  |  |
| How do you treat the alpha-gal/red meat allergy? | | Antibiotics | 4 (5.6) | There is no therapy | 34 (47.2) |
|  |  | Monoclonal Ab therapy | 1 (1.4) |  |  |
|  |  | Antigen stimulus therapy | 2 (2.8) |  |  |
|  |  | No answer | 31 (43.1) |  |  |

**Supplemental Table 2:** Responses to questions about education, outreach, and training around ticks and tick-borne diseases among veterinary professionals in Illinois

| Question | | Yes (%) | No (%) | No Answer (%) |
| --- | --- | --- | --- | --- |
| Do you feel that client education about ticks and tick-borne diseases is important? | | 48 (66.7) | 0 (0) | 24 (33.3) |
| Do you think that tick-borne diseases are a concern in your area? | | 44 (61.1) | 5 (6.9) | 23 (31.9) |
| Would you like to have training on ticks and tick-borne diseases? | | 30 (41.7) | 18 (25.0) | 24 (33.3) |
| What tick and tick-borne disease topics would you like further  training in? | Tick Species | 24 (33.3) | 48 (66.7) |  |
|  | Tick Removal | 5 (6.9) | 67 (93.1) |  |
|  | Tick-borne diseases in your area | 31 (43.1) | 41 (56.9) |  |
|  | Testing for tick-borne diseases | 27 (37.5) | 45 (62.5) |  |
|  | Treatment of tick-borne diseases | 31 (43.1) | 41 (56.9) |  |
|  | Community outreach/communication about ticks and diseases | 19 (26.4) | 53 (73.6) |  |
|  | Other | 1 (1.4) | 69 (95.8) | 2 (2.8) |
| Do you feel like more public outreach about ticks and tickborne diseases from the Illinois Department of Public Health (IDPH), American Veterinary Medical Association (AVMA), or the University of Illinois would benefit your area? | | 41 (56.9) | 8 (11.1) | 23 (31.9) |
| Who would you like to receive public outreach from? | IDPH | 21 (29.2) | 51 (70.8) |  |
|  | AVMA | 21 (29.2) | 51 (70.8) |  |
|  | UIUC | 29 (40.3) | 43 (59.7) |  |

**Supplemental Table 3:** Responses to questions about practices regarding ticks and tick-borne diseases among veterinary professionals in Illinois

| Question | | Yes (%) | No (%) | No Answer (%) |
| --- | --- | --- | --- | --- |
| Do you routinely look for ticks on yourself and/or patients? | | 43 (59.7) | 6 (8.3) | 23 (31.9) |
| Do you routinely ask about tick exposure when talking to clients? | | 40 (55.6) | 7 (9.7) | 25 (34.7) |
| What samples do you prefer to use/submit? | Serum | 20 (27.8) | 52 (72.2) |  |
|  | Blood | 39 (54.2) | 33 (45.8) |  |
|  | Joint Fluid | 0 (0) | 72 (100.0) |  |
|  | Other | 0 (0) | 70 (97.2) | 2 (2.8) |
| When you have a patient present with signs of a tick-borne disease like a fever of unknown origin, joint pain, and lethargy, do you ask about travel history? | | 24 (33.3) | 21 (29.2) | 27 (37.5) |
| Do you provide information about tick-borne diseases to your clients on a routine basis? | | 27 (37.5) | 21 (29.2) | 24 (33.3) |
| If you do provide information to your clients about tick-borne  diseases, how do you do this? | Flyers/Pamphlets | 21 (29.2) | 51 (70.8) |  |
|  | Verbally | 32 (44.4) | 40 (55.6) |  |
|  | Refer to a website | 3 (4.2) | 69 (95.8) |  |
|  | Other | 0 (0) | 70 (97.2) | 2 (2.8) |
| Question | **Response** | | | **N (%)** |
| When do you test for tick-borne diseases? | Routinely | | | 20 (27.8) |
|  | When suspecting a tick-borne disease | | | 22 (30.6) |
|  | To rule out a tick-borne disease | | | 4 (5.6) |
|  | No answer | | | 26 (36.1) |
| What is the main reason clients decline testing for tick-borne diseases? | They find it unnecessary | | | 10 (13.9) |
|  | Cost is a limiting factor | | | 23 (31.9) |
|  | Tick-borne disease is not a main differential | | | 8 (11.1) |
|  | Other | | | 4 (5.6) |
|  | No Answer | | | 27 (37.5) |

**Supplemental Table 4:** Survey Responses on experiences with ticks and tick-borne diseases

| Question | | *Yes (%)* | *No (%)* | *No Answer (%)* |
| --- | --- | --- | --- | --- |
| Please indicate tick-borne diseases for which you have had patients test positive in the past two years | Anaplasmosis | 25 (34.7) | 47 (65.3) |  |
|  | Babesiosis | 4 (5.6) | 68 (94.4) |  |
|  | *Borrelia miyamotoi* | 0 (0) | 72 (100.0) |  |
|  | Lyme disease | 39 (54.2) | 33 (45.8) |  |
|  | Bourbon virus | 0 (0) | 72 (100.0) |  |
|  | Ehrlichiosis | 37 (51.4) | 35 (48.6) |  |
|  | Heartland virus | 1 (1.4) | 71 (98.6) |  |
|  | Powassan disease | 0 (0) | 72 (100.0) |  |
|  | Rickettsiosis | 2 (2.8) | 70 (97.2) |  |
|  | Rocky Mountain Spotted Fever | 7 (9.7) | 63 (87.5) | 2 (2.8) |
|  | STARI | 0 (0) | 67 (93.1) | 5 (6.9) |
|  | Tularemia | 1 (1.4) | 64 (88.9) | 7 (9.7) |
| Have you removed any of these ticks from patients? | *Ixodes scapularis* | 29 (40.3) | 43 (59.7) |  |
|  | *Rhipicephalus sanguineus* | 32 (44.4) | 40 (55.6) |  |
|  | *Amblyomma maculatum* | 1 (1.4) | 71 (98.6) |  |
|  | *Amblyomma americanum* | 24 (33.3) | 48 (66.7) |  |
|  | *Dermacentor variabilis* | 28 (38.9) | 44 (61.1) |  |
|  | *Dermacentor albipictus* | 2 (2.8) | 70 (97.2) |  |
| *Question* | | ***Mean (s.d.)*** | | |
| How many cases of these diseases have you diagnosed in the last two years? | Anaplasmosis | 11.04 (22.30) | | |
|  | Babesiosis | 1.00 (0.00) | | |
|  | *Borrelia miyamotoi* | NA | | |
|  | Lyme disease | 13.61 (24.45) | | |
|  | Bourbon virus | NA | | |
|  | Ehrlichiosis | 20.65 (30.45) | | |
|  | Heartland virus | 0 (NA) | | |
|  | Powassan disease | NA | | |
|  | Rickettsiosis | 1.00 (NA) | | |
|  | Rocky Mountain Spotted Fever | 1.38 (1.77) | | |
|  | STARI | NA | | |
|  | Tularemia | 1 (1.4) | | |
| Knowledge score | | 22.81 (10.78) | | |
| Tick-specific knowledge score | | 15.75 (7.22) | | |
| Tick-borne disease-specific knowledge score | | 7.06 (3.76) | | |
